# Novel investigation of perovskite membrane based electrochemical nitric oxide control phenomenon

**DOI:** 10.1038/s41598-020-75360-7

**Published:** 2020-10-30

**Authors:** Thomas S. Welles, Jeongmin Ahn

**Affiliations:** Department of Mechanical and Aerospace Engineering, Syracuse University, Syracuse, NY 13244-1240 USA

**Keywords:** Catalysis, Electrochemistry, Environmental chemistry, Surface chemistry, Aerospace engineering, Mechanical engineering, Materials for devices

## Abstract

The combustion of hydrocarbon fuels within the automotive industry results in harmful and reactive incomplete combustion byproducts. Specifically, nitric oxide emissions (NO) lead to increased smog, acid rain, climate change, and respiratory inflammation within the population [Nitrogen Dioxide | American Lung Association]. Current methods for treating combustion exhaust include the catalytic converter in conjunction with nitrogen oxide traps. However, there is no active, continuous reduction method that does not require restrictions on the combustion environment (Hirata in Catal Surv Asia 18:128–133, 2014). Here, a small voltage potential oscillation across a newly designed electro-chemical catalytic membrane significantly reduces NO emissions. A ceramic membrane consisting of two dissimilar metal electrodes, sandwiching a dielectric layer, is able to achieve an NO reduction in excess of 2X that of a platinum group metal (PGM) three way catalytic converter. An analysis of the exhaust effluent from the membranes indicates N_2_O as a precursor to N_2_ and O_2_ formation, without the introduction of ammonia (NH_3_), during the reaction of NO indicating a divergence from current literature. Our results demonstrate how an oscillatory electric potential on a catalytic surface may alter anticipated reaction chemistry and interaction between the catalytic surface and fluid flow.

## Introduction

**Figure 1:**
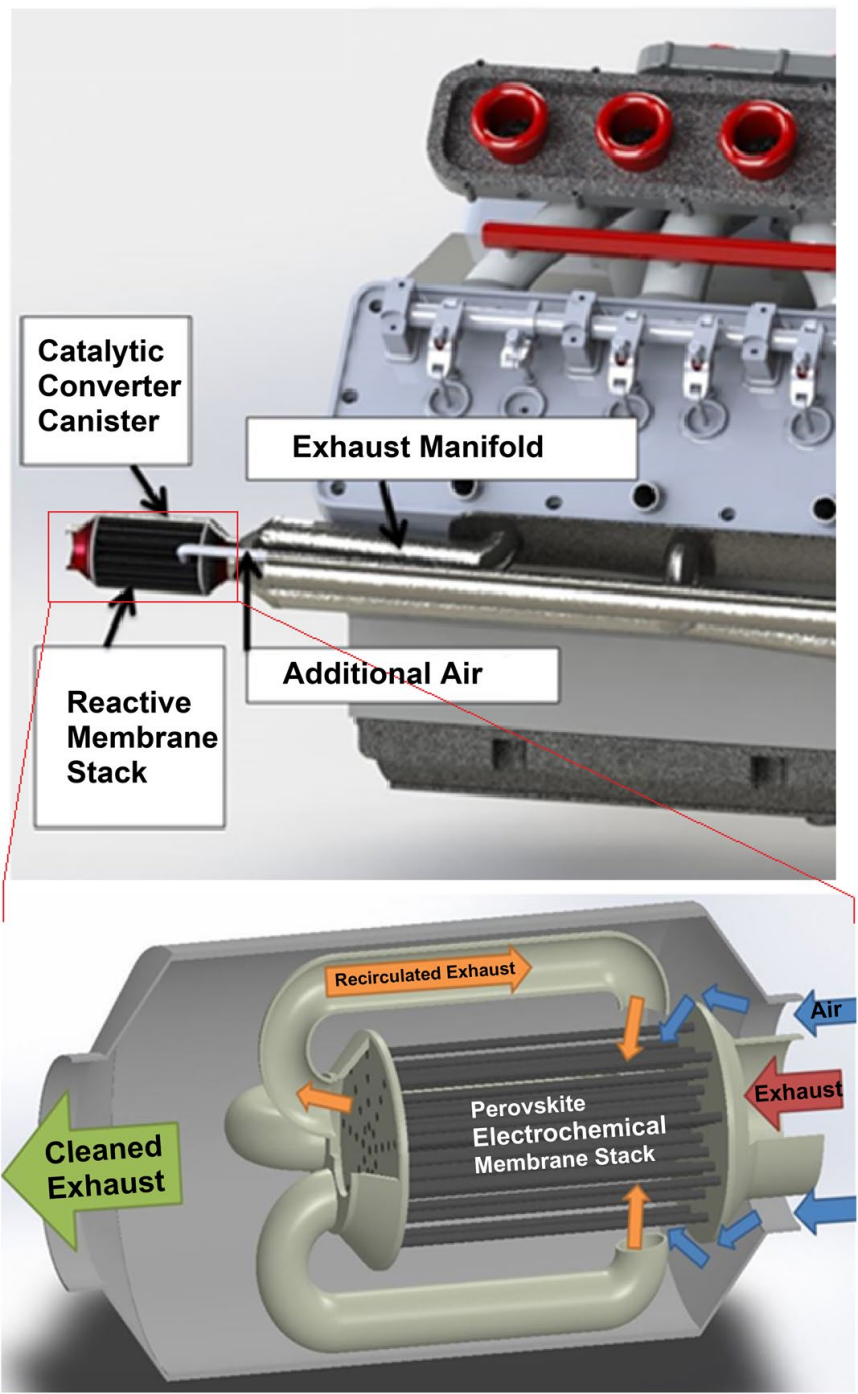
Schematic of electrochemical catalytic membrane integration into automotive exhaust. Patent Pending: US 2020–0,052,316 A1.

Increased concern over climate change, harmful combustion emissions and poor air quality has created a greater need for efficient catalytic emission processing systems^1–3^. Traditionally, three-way catalysts, such as those found in automobiles, and nitrogen oxide traps have been employed to reduce pollutants such as hydrocarbons, carbon monoxide and nitric oxide (NO). However, the performance of these devices is highly dependent upon the equivalence ratio of the exhaust. Three-way catalysts require that the exhaust remain at stoichiometric conditions^4–6^. If the exhaust becomes fuel lean, a nitrogen oxide trap is required, which will eventually become clogged with NO emission, calling for the direct injection of a fuel into the trap in order to react the stored gas^6–16^. A new technology must be developed that can more efficiently reduce harmful emissions while continuously operating in any exhaust condition. Therefore the potential of a layered perovskite membrane is investigated as an alternative catalyst to platinum group metals (PGM). The electrochemical catalytic membrane stack will be placed just downstream of the exhaust manifold of the engine. If needed, additional air may be injected into the housing canister in the case of a rich combustion environment during startup. The combustion engine’s exhaust gases will then flow through the first tubular support layer of the membrane stack, as illustrated by Fig. 1.

The tubular support layer is tuned to primarily convert unburned hydrocarbons, H_2_, and CO into water and carbon dioxide. The exhaust gas, upon exiting the tubular first layer, will be recycled to the outer perovskite layer of the membrane. This layer will then be primarily focused on the reaction of NO into N_2_ and O_2_. The support and outer layer will be separated by a dielectric ceramic layer. The exhaust gas will then leave the electrochemical catalytic stack, continuing within the automotive exhaust system.

The initial work was designed to directly compare the potential of the electrochemical-catalytic membrane for emission control against a traditional PGM catalytic converter. The PGM based catalytic converter is a commercially available catalytic converter purchased from the Volkswagen-Audi Group (VAG). It is a modern three-way catalyst, with an internal honeycomb structure consisting of a platinum combined with palladium and rhodium. Each specimen was held at 600 °C and subjected to 10 ml/min of two extremely lean (excessive O_2_ and limited hydrocarbon presence) model combustion exhausts, creating the most challenging environment for the electrochemical-catalytic membrane, and one stoichiometric exhaust, utilizing CH_4_ as a model hydrocarbon component of the exhaust. Although modern internal combustion engine control systems would not allow for such extreme conditions, the test is of academic interest to investigate how catalysts perform beyond normal operating conditions. The model exhausts were created by combining O_2_, CH_4_, CO, CO_2_, N_2_, and NO via benchtop flow meters.

The results shown below in Table 1 represent the time averaged results of each specimen. The specimen was introduced into the furnace 60 min prior to the collection of any effluent, for analysis to allow each sample to obtain steady state operation. The effluent of each sample specimen was then collected, analyzed, and recorded by the E-Instruments emission analyzer and gas chromatography-mass spectroscopy (GC–MS). The results were averaged over a constant 3 h test for each specimen.

**Table 1:** Lean Emission Reduction Comparison of the Electrochemical-Catalytic Membrane and the Catalytic Converter at 600 °C^17^.

	O_2_%	CO %	CO_2_%	NO ppm	NO_2_ ppm	C_x_H_y_ ppm
**Lean Mixture A**						
Baseline	8	2.2	22.9	46	0	6725
Catalytic Converter	7.57	1.36	23.6	39.17	1.83	6550
Electrochemical-Catalytic Membrane	7.11	0.59	24.6	34.5	0	6656
**Lean Mixture B**						
Baseline	11.3	3	22.1	125	0	3200
Catalytic Converter	10.9	2.79	23	122.8	0	3141
Electrochemical-Catalytic Membrane	6.15	2.44	26.1	72.25	3	2615
**Stoichiometric Mixture C**						
Baseline	18.31	3.05	0	760	0	9.14
Catalytic Converter	17.21	1.64	1.67	705	0	8.84
Electrochemical-Catalytic Membrane	16.89	2.01	1.24	601	2	8.91

The electrochemical-catalytic membrane is able to significantly outperform the PGM catalytic converter in the reduction of NO and CO, and closely matches the PGM catalytic converter in the reduction of hydrocarbons. Additionally, the electrochemical-catalytic membrane is able to maintain function in the presence of high concentrations of oxygen. The ability of the electrochemical-catalytic converter to maintain reactivity of NO at varying levels of NO has been previously reported^17^.

The novel electrochemical-catalytic membrane is able to consistently outperform traditional catalytic membranes for continuous NO reduction across multiple operation conditions. However, during testing, a minute oscillatory electrical voltage was seen to develop across the membrane.

Therefore, this work is designed to investigate the fundamental relation between electrical activity and reaction mechanism by which the novel electrochemical-catalytic membrane is able to react NO.

Catalytic perovskite materials are arranged such that two metal-based catalytic layers are separated by and in contact with a dielectric membrane for the study of NO reduction. The resulting electrochemical catalytic membrane is subjected to model exhaust flows within a controlled laboratory to serve as an initial investigation into the emission control potential.

## Materials and methods

The support layer is comprised of nickel oxide-gadolinium doped ceria (48.9 vol% NiO 51.1 vol% Gd_0.10_Ce_0.90_O_2-x_ (NiO-GDC)), the dielectric material of gadolinium doped ceria (Gd_0.10_Ce_0.90_O_1.95_ (GDC)) and the outer layer of lanthanum strontium cobalt ferrite-gadolinium doped ceria (52.4 vol% (La_0.60_Sr_0.40_)_0.95_Co_0.20_Fe_0.80_O_3-X_ 47.6 vol% Gd_0.10_Ce_0.90_O_1.95_ (LSCF-GDC))^18–23^. Although these materials are commonly utilized in the production of high temperature solid oxide fuel cells, their purpose here is to act only as a catalytic membrane. As such, the nickel oxide was not reduced to nickel prior to testing. Pure silver wire electrodes were then added to both the support and outer layers via silver paste at the edge of the layer.

A test was constructed in order to investigate the fundamental mechanism by which the cathode layer of the electrochemical-catalytic membrane was able to significantly breakdown NO. The new electrochemical-catalytic membrane, with 0.81 cm^2^ of reactive surface area, is tested for NO reduction against: 1. VAG catalytic converter with 0.81 cm^2^ of reactive surface area; 2. The same electrochemical-catalytic membrane with the anode and cathode wired together to resemble a short circuit condition (hereafter referred to as an external short circuit or short circuited); and 3. The same electrochemical-catalytic membrane with an external 3 V, 8000 μs (4% duty cycle) pulse width modulation (PWM) signal with 0.81 cm^2^ of reactive surface area.

Each specimen was held at 600 °C and subject to 5 ml/min flow of a certified premixed gas cylinder of 10 vol% NO and 90 vol% N_2_. All testing equipment was cleaned, dried, and flushed with Ar for 1 h within the furnace at 600 °C prior to testing, to ensure no contamination of results. Only N_2_ and NO were supplied to the testing apparatus. During each test, the internal furnace environment surrounding the testing equipment was flooded with Ar, in order to ensure an external inert environment and to detect any leakage. For the electro-chemical catalyst, the NO flow was sent to the cathode side of the membrane. The voltage signal for both the electro-chemical catalytic membrane and the short-circuited membrane were recorded with a 100 MHz oscilloscope.

**Figure 2:**
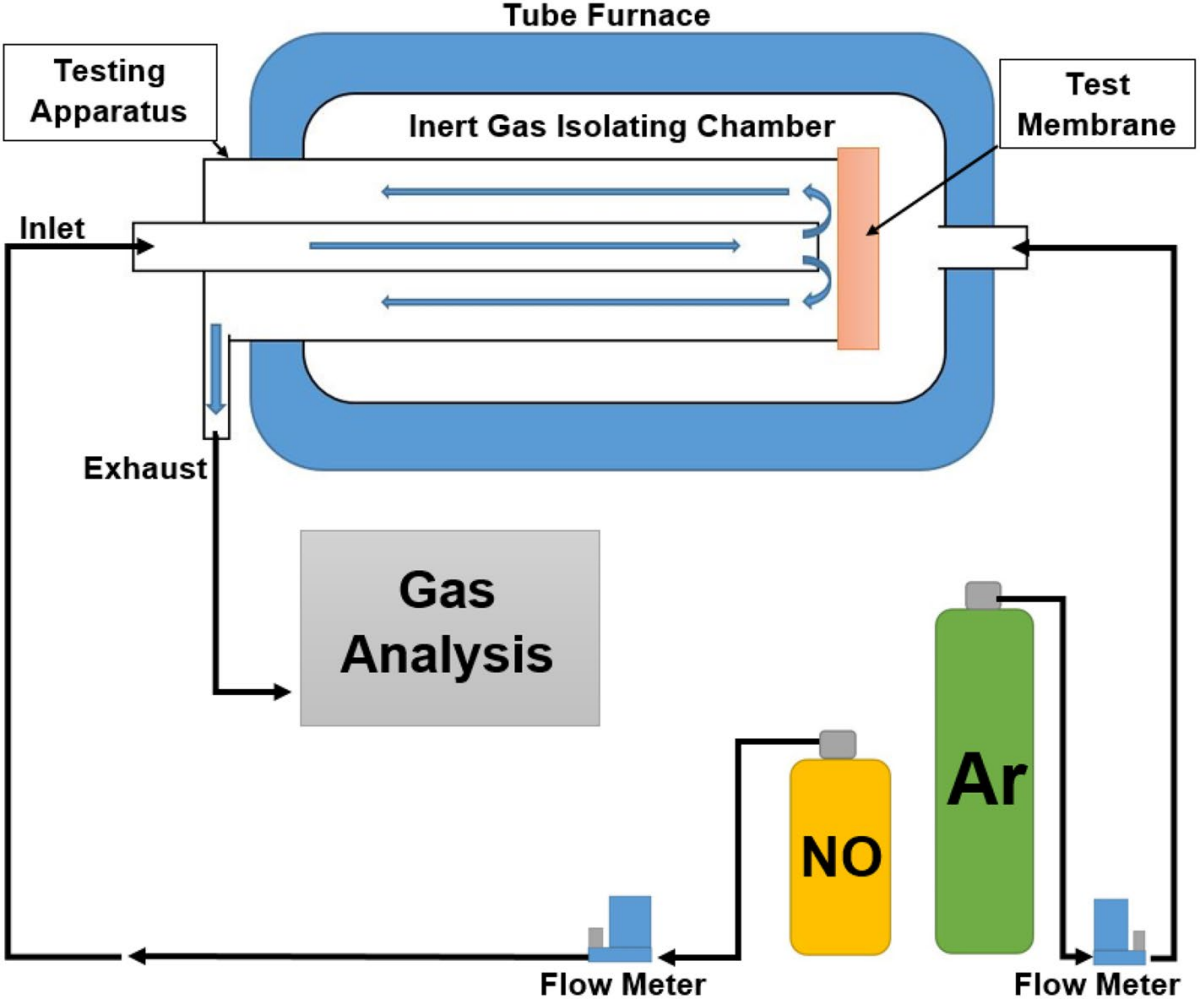
Schematic of membrane testing apparatus.

For both of the experimental setups, the effluent of each specimen, recorded with a Hiden Analytic QGA MS with a 100 ppb detection limit, verified via GC by a SRI 8610C and an E-instruments E-8500 emission analyzer, is compared to the baseline effluent. The experimental setup for the second, one sided test is depicted below in Fig. 2. The experimental setup for the initial study is the same, except before the effluent leaves the testing chamber it is recycled over the opposite side of the test specimen and then is exhausted.

## Results and discussion

### Experimental results of NO reduction phenomenon

The electro-chemical catalytic membrane, consisting of two metal electrode surfaces separated by a dielectric electrolyte, was able to achieve a reduction in NO concentration greater than 2X that of a traditional PGM catalyst as seen in Table 2 above. Additionally, wiring an external oscillating voltage signal between the two metal electrodes of the membrane increased reduction potential by an additional 10%. This behavior is in stark contrast to that of the electro-chemical catalytic membrane with the addition of an external short circuit, which was only able to convert an amount of NO similar to a traditional PGM catalyst.

**Table 2:** Experimental Results: NO Reduction Comparison of the Electro-chemical Catalytic Membranes and Traditional Catalyst Specimens.

Specimen Sample	NO Concentration in Effluent (vol %)	Percent Reduction from Baseline (%)
Baseline	10.0	–
PGM Catalytic Converter	7.08	29.2
Short Circuited Electro-chemical Catalytic Membrane	7.01	29.9
Electro-chemical Catalytic Membrane	3.10	69.0
Electro-chemical Catalytic Membrane with 3 V PWM Signal	2.07	79.3

**Figure 3:**
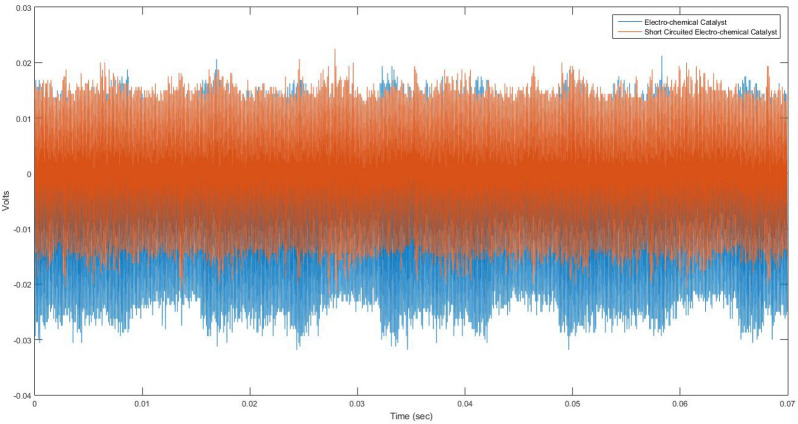
Electrical Signal Recording Overlays of the Novel Electrochemical Catalytic Membrane with (shown in orange) and without (shown in blue) an External Electrical Short Circuit at 600 °C and Subjected to 5 ml/min Flow of a Certified Premixed Gas Cylinder of 10 vol% NO and 90 vol% N_2_. Signal Noise Amplitude of ~ 35 mV with Electrical Short (orange) and ~ 50 mV without the Short Circuit (blue).

Figure 3 shows the comparison of the voltage signal generated by the electro-chemical catalytic membrane and the electro-chemical catalytic membrane with an external short circuit during the NO reduction. The externally short-circuited membrane is centered on a voltage potential of 0, whereas the membrane without a short circuit is centered around -6.0 mV, under the standard convention of the cathode being the electrically positive electrode.

Additionally, the oscillations of the membrane without a short circuit are 2X the magnitude of those seen in the short-circuited membrane. Amplification of voltage oscillations within the membrane, as with the addition of a PWM external source, increases the total amount of NO reduction. Therefore, the ability of the electro-chemical catalytic membrane to charge and discharge while interacting with an external flow is the key difference allowing for significantly increased reaction rates.

The charge, discharge behavior of the electro-chemical catalytic membrane significantly differs from the reaction pathway previously accepted in literature.

**Figure 4:**
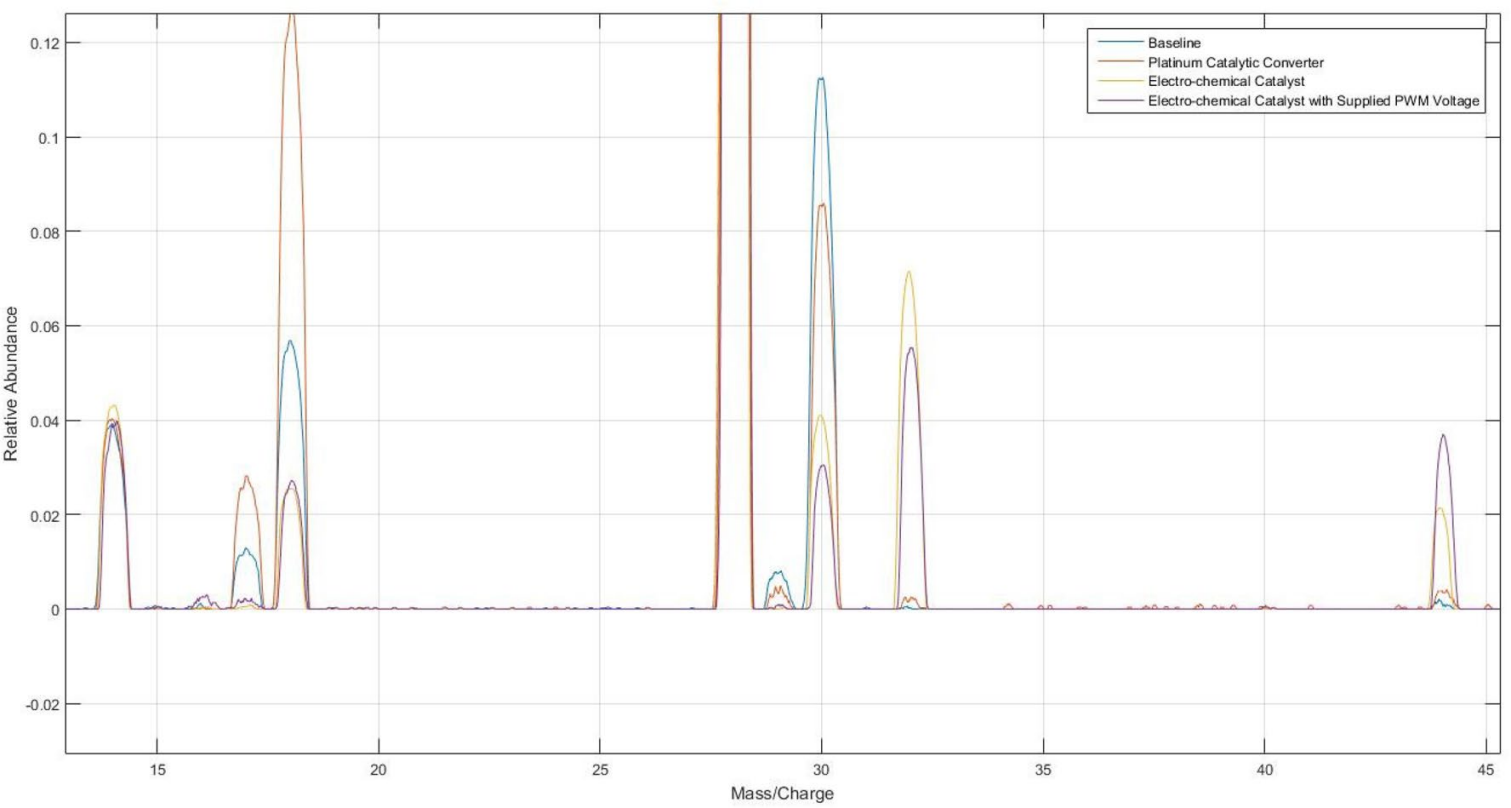
Mass Spectrum Analysis Comparison of Exhaust Effluent after Interacting with Each Specimen at 600 °C and Subject to 5 ml/min Flow of a Certified Premixed Gas Cylinder of 10 vol% NO and 90 vol% N_2_. Baseline Represents the Effluent of the Same Gas through the Testing Apparatus at Temperature without Any Reactive Membrane in Place. **The electro-chemical catalytic membrane with external short circuit is not shown, as it nearly coincides with the traditional PGM catalytic converter, reducing the clarity of the plot.

The electro-chemical catalytic membrane shows significant deviations from all other specimens in the mass spectrum at 17, 18, 30, 32 and 44 amu, as shown in Fig. 4. The difference at 30 and 32 amu represent a reduction in NO and production of O_2,_ respectively. The large peak at 18 amu, followed by sub-peaks at 17 and 16 amu indicates lone and charged oxygen. The peak at 44 amu indicates a concentration of N_2_O. The decrease in magnitude at 18 amu and the substantial peak at 44 amu represents a significant deviation from the majority of literature for NO breakdown. NO reduction has traditionally been separated into two categories: 1. Chemical gradient and equilibrium with or without a catalyst present^24,28^; 2. Combustion reaction^29–32^.

A high concentration of NO will drive an equilibrium reaction given sufficient energy and/or the presence of a catalyst^31–33^.

$${N}_{2}+{O}_{2}\leftrightarrow {N}_{2}{O}_{2}\leftrightarrow 2 NO$$

NO may also undergo a combustion reaction with ammonia (NH_3_), air and/or hydrocarbons (C_x_H_y_) in which NO will typically pass through an intermediary of NO_2_^29,34–38^.

In this study, there is clear formation of N_2_O without additional reactants being sourced to aid the reduction of NO. Previously, an intermediary of N_2_O during the breakdown of NO had only been recorded while supplying ammonia to a platinum catalyst at 850 °C^29,31–37^. It is therefore believed that the electrical oscillation activity of the electro-chemical membrane is the reason for an altered reaction pathway and significant reduction of NO. This hypothesis is primarily investigated through a Gibbs free energy analysis. The following table lists potential reactions and respective changes in Gibbs free energy for a temperature range near the testing point of 600 °C in the breakdown of NO and eventual formation of N_2_ and O_2_.

For simplicity of analysis, it is assumed that the first step in the breakdown of NO is the formation of N_2_O_2_, as has been reported previously in literature^17–27,31–42^. Therefore, the key equations to investigate in Table 3, given sufficient concentration of N_2_O_2_, are reaction equations numbers 8 and 10. It is clear that at 600 °C the formation of NO_2_ is energetically favorable to the formation of NO_2_, reaction 10, if the Gibbs free energy minimization is the only driving factor. However, it also indicates that the formation of N_2_O, reaction 8, could also be spontaneous but is less probable to occur than reaction 10. Therefore, it is reasonable to assume that an electrochemical manipulation/influence could drive the likelihood of occurrence toward reaction 8 and the formation of N_2_O.

**Table 3:** Gibbs free energy of reaction analysis summary of potential chemical reaction equations.

Gibbs Free Energy Analysis of Reaction (kJ)									
Chemical Reaction		Temperature of Reaction (°C)							
		450	500	550	600	650	700	750	800
1	${N}_{2}+{O}_{2}\to {N}_{2}{O}_{2}$	219.5	225.0	230.4	235.9	241.3	246.8	252.3	257.7
2	${N}_{2}+{O}_{2}\to 2NO$	169.4	168.2	166.9	165.7	164.4	163.2	161.9	160.7
3	$2NO\to {N}_{2}{O}_{2}$	50.1	56.8	63.5	70.2	76.9	83.6	90.3	97.0
4	${N}_{2}+{O}_{2}\to N{O}_{2}+N$	507.4	507.6	507.7	507.9	508.0	508.2	508.4	508.5
5	${N}_{2}+{O}_{2}\to {N}_{2}O+O$	338.4	339.2	339.9	340.7	341.5	342.3	343.1	343.9
6	${N}_{2}+{O}_{2}\to N{O}_{2}+\frac{1}{2}{N}_{2}$	60.6	63.6	66.6	69.7	72.7	75.8	78.8	81.8
7	${N}_{2}+{O}_{2}\to {N}_{2}O+\frac{1}{2}{O}_{2}$	115.5	119.2	122.9	126.7	130.4	134.1	137.8	141.5
8	${N}_{2}{O}_{2}\to {N}_{2}O+\frac{1}{2}{O}_{2}$	− 104.0	− 105.7	− 107.5	− 109.2	− 111.0	− 112.7	− 114.5	− 116.2
9	${N}_{2}{O}_{2}\to {N}_{2}O+O$	118.9	114.2	109.5	104.8	100.2	95.5	90.8	86.1
10	${N}_{2}{O}_{2}\to N{O}_{2}+\frac{1}{2}{N}_{2}$	− 158.9	− 161.4	− 163.8	− 166.2	− 168.6	− 171.0	− 173.5	− 175.9
11	${N}_{2}{O}_{2}\to N{O}_{2}+N$	287.9	282.6	277.3	272.0	266.7	261.4	256.1	250.8
12	$2{N}_{2}O\to 2{N}_{2}+{O}_{2}$	− 231.0	− 238.5	− 245.9	− 253.3	− 260.7	− 268.2	− 275.6	− 283.0
13	${N}_{2}O+NO\to \frac{3}{2}{N}_{2}+{O}_{2}$	− 200.2	− 203.3	− 206.4	− 209.5	− 212.6	− 215.7	− 218.8	− 221.8
14	$2N{O}_{2}\to {N}_{2}+2{O}_{2}$	− 121.1	− 127.2	− 133.3	− 139.4	− 145.4	− 151.5	− 157.6	− 163.7
15	$N{O}_{2}+NO\to {N}_{2}+\frac{3}{2}{O}_{2}$	− 145.3	− 147.7	− 150.1	− 152.5	− 154.9	− 157.3	− 159.8	− 162.2
16	${N}_{2}O+NO\to {N}_{2}+N+{O}_{2}$	246.6	240.7	234.7	228.7	222.8	216.8	210.8	204.9
17	$N{O}_{2}+NO\to {N}_{2}+{O}_{2}+O$	77.6	72.3	66.9	61.6	56.2	50.9	45.5	40.2

Additionally, the transfer efficiency from NO to N_2_ is considered in conjunction with the Gibbs free energy analysis. Table 3 indicates in reactions 12 and 14 that N_2_O as an intermediary species should result in a higher yield of N_2_ when compared to NO_2._

Table 4 above calculates the transfer efficiency factor from NO reduction to N_2_ production. The ideal increase in N_2_ is calculated from the reduction of NO reported in Table 2, and represents the amount of N_2_ that could be produced if the transfer efficiency were equal to 1. That is, if all NO reduction resulted in the formation of N_2_. It is clear to see that the electrochemical catalytic membrane with enhanced electrical activity has a significantly better transfer efficiency at 0.94. There is a direct correlation between N_2_O production and increased N_2_ transfer efficiency. The negative factor for the PGM catalytic converter is not considered to be abnormal for catalytic converter operation. It has been reported that commercial PGM catalytic converters do have limited N_2,_ NO storage capability, which would account for the discrepancy in N_2_ concentration in the exhaust^4–12^.

**Table 4:** Experimental Results: N_2_ Production Comparison of the Electrochemical Catalytic Membranes and Traditional Catalyst Specimens with Transfer Efficiency Factors Calculated.

Specimen Sample	N_2_ Concentration in Effluent (vol %)	Change from Baseline (vol %)	Ideal N_2_ Increase from Baseline based on NO Reduction (vol %)	Transfer Efficiency Factor
Baseline	90.0	–	–	–
PGM Catalytic Converter	84.62	− 5.38	1.46	− 3.69
Electro-chemical Catalytic Membrane with short circuit	90.88	0.88	1.50	0.59
Electro-chemical Catalytic Membrane	92.51	2.51	3.45	0.73
Electro-chemical Catalytic Membrane with 3 V PWM Signal	93.72	3.72	3.97	0.94

Formation of N_2_O is seen in the mass spectrum analysis of the electrochemical catalytic membrane and more predominately in the electrochemical catalytic membrane with forced electrical oscillations. It is not as evident in the effluent of the traditional PGM catalyst, nor the electrochemical catalytic membrane with an electrical connection between the anode and cathode. Therefore, when the electrical activity on the membrane is not present, the formation of N_2_O is significantly diminished.

The deposition, removal, and rearrangement of electrons from/on the catalytic surface is believed to be the key in altering the probability of reaction pathways. Traditional catalytic reaction chemistry with a solid, non-consumable catalyst without the presence or possibility of water formation or the presence of a proton acceptor/donor, assumes that the catalytic surface, after absorbing the reactant, acts primarily as an electron transport mechanism around the reactant molecule^43,44^. For the above experimental results, the apparatus was free of any water vapor, as described in the methods section, and the test flow consisted of only nitrogen and oxygen, eliminating the possibility of acid–base catalysis. Within any catalyst in which there is electron movement around the reactant, there must be an ohmic like resistance. However, if the charge on the catalytic surface is forced to vary, i.e. the surface is forced to charge and discharge, electron movement to and from the surface is established prior to the absorption of the reactant molecule. The addition and removal behavior of electrons that is established on the surface is believed to manipulate the natural reaction pathways increasing the overall reactivity of the system. In addition, this alternating voltage potential creates a fluctuating external electric field capable of doing work and exciting any incoming polar molecule. The external field may also manipulate the collisions of the incoming reacting species by forcing varying molecular alignment as the flow approaches the catalytic surface.

## Conclusions

The unique configuration of a metal-based catalytic surface connected through a dielectric membrane to another metal-based electrode allows for the potential of surface charging and charge communication without significant internal current flow or internal charge balancing. This charging and discharging of the catalytic surface allows for the altered chemical reaction pathways demonstrated in this work. When the two metal electrodes of the electro-chemical catalytic membrane are electrically connected, the reactivity decreased by a factor of ~ 2. Any charge that began to develop on the surface was able to relax back into equilibrium via current flow. Therefore, the surface charge was no longer capable of reacting with the incoming flow, decreasing efficacy of NO conversion.

Further investigation into this phenomenon has led to the consideration of CO and CO_2_ conversion to particulate carbon and gaseous oxygen. Although this exploration is in its infancy, it does indicate potential for the electrochemical manipulation of carbon based emissions through electric potential oscillation. However, unlike the work described in the background of this work^17^, initial testing has shown significant carbon deposition and carbon coking of the electrode layer during prolonged operation in a purely CO and CO_2_ environment, decreasing reactivity over time. Additional work is required to develop a technology capable of continually reacting CO and CO_2_ into particulate carbon and gaseous oxygen without the addition of other reactants to prevent coking.
